# Ultra-low-dose sequential computed tomography for quantitative lung aeration assessment—a translational study

**DOI:** 10.1186/s40635-017-0133-6

**Published:** 2017-04-04

**Authors:** Lorenzo Ball, Anja Braune, Francesco Corradi, Claudia Brusasco, Alessandro Garlaschi, Thomas Kiss, Thomas Bluth, Francesca Simonassi, Alice Bergamaschi, Jörg Kotzerke, Marcus J. Schultz, Marcelo Gama de Abreu, Paolo Pelosi

**Affiliations:** 1 Department of Surgical Sciences and Integrated Diagnostics, University of Genoa, Largo Rosanna Benzi 8, Genoa, Italy; 2 Department of Radiology, IRCCS AOU San Martino-IST, Genova, Italy; 3 Department of Anesthesia and Intensive Care, Ente Ospedaliero Galliera, Genova, Italy; 4 Department of Intensive Care, Academic Medical Center, University of Amsterdam, Amsterdam, Netherlands; 5 Department of Anesthesiology and Intensive Care Medicine, University Hospital Carl Gustav Carus, Dresden, Germany; 6 Institute of Nuclear Medicine, Technische Universität Dresden, Dresden, Germany

**Keywords:** Computed tomography, Radiation dose, Lung injury

## Abstract

**Background:**

Quantitative lung computed tomography (CT) provides fundamental information about lung aeration in critically ill patients. We tested a scanning protocol combining reduced number of CT slices and tube current, comparing quantitative analysis and radiation exposure to conventional CT.

**Methods:**

In pigs, CT scans were performed during breath hold in a model of lung injury with three different protocols: standard spiral with 180 mAs tube current-time product (Spiral180), sequential with 20-mm distance between slices and either 180 mAs (Sequential180) or 50 mAs (Sequential50). Spiral scans of critically ill patients were collected retrospectively, and subsets of equally spaced slices were extracted. The agreement between CT protocols was assessed with Bland–Altman analysis.

**Results:**

In 12 pigs, there was good concordance between the sequential protocols and the spiral scan (all biases ≤1.9%, agreements ≤±6.5%). In Spiral180, Sequential180 and Sequential50, estimated dose exposure was 2.3 (2.1–2.8), 0.21 (0.19–0.26), and 0.09 (0.07–0.10) mSv, respectively (*p* < 0.001 compared to Spiral180); number of acquired slices was 244 (227–252), 12 (11–13) and 12 (11–13); acquisition time was 7 (6–7), 23 (21–25) and 24 (22–26) s. In 32 critically ill patients, quantitative analysis extrapolated from 1-mm slices interleaved by 20 mm had a good concordance with the analysis performed on the entire spiral scan (all biases <1%, agreements ≤2.2%).

**Conclusions:**

In animal CT data, combining sequential scan and low tube current did not affect significantly the quantitative analysis, with a radiation exposure reduction of 97%, reaching a dose comparable to chest X-ray, but with longer acquisition time. In human CT data, lung aeration analysis could be extrapolated from a subset of thin equally spaced slices.

**Electronic supplementary material:**

The online version of this article (doi: 10.1186/s40635-017-0133-6) contains supplementary material, which is available to authorized users.

## Background

The quantitative analysis of lung computed tomography (CT) has profoundly changed the understanding of acute respiratory distress syndrome (ARDS) [1–4]. Despite the emergence of bedside tools for the assessment of lung aeration such as lung ultrasound and electric impedance tomography, CT is still the gold standard [5]. In fact, CT is the only imaging technique showing a direct correlation with density [6], from which lung aeration can be inferred with a widely accepted model assuming lung weight as a linear function of Hounsfield units (HU) [7]. Lung aeration compartments can be calculated as percent of the total lung weight, and normal values of healthy adults were recently published [8].

Translation from the research setting to clinical practice of this technique is significantly hampered by practical issues; apart from the need to move the patient from the intensive care unit (ICU) to the CT facility, the necessary high ionizing radiation exposure, and long post-processing segmentation times are important hurdles. To overcome part of these hampering factors, low-dose spiral CT acquisition protocols have been proposed [9, 10]. These methods, while reducing radiation dose, do not reduce the time needed to perform lung segmentation. So far, the only validated means to reduce post-processing time have been visual anatomical estimation of lung aeration [9, 11] or the extrapolation of a reduced subset of ten slices from a whole-chest spiral CT [12–14].

Sequential CT is an acquisition mode in which axial slices are acquired interleaved by an arbitrary distance (feed) along which the patient is not irradiated. Although replaced by spiral volumetric scan modes in most routine CT studies, sequential acquisition is still available in modern scanner because of its superiority in specific indications [15] and has been proposed in lung imaging to reduce dose exposure [16].

The aim of the present study was to investigate the accuracy and feasibility of a lung CT scanning protocol combining the prospective acquisition of a reduced number of thin slices with a reduced tube current, and to estimate the resulting radiation dose and segmentation time reduction. We hypothesized that low-dose sequential CT scan does not result in major loss of information on lung aeration while reducing exposure to radiation compared to conventional CT. For this purpose, we chose a translational study design: in the first part, the method was tested in an animal model of ARDS, and in the second part its robustness was tested in a cohort of critically ill patients.

## Methods

In the first part of this investigation, CT scans performed in a porcine model of lung injury (location: university research facility in Dresden, Germany) were analyzed. In the second part, retrospectively collected chest spiral CT scans of patients in the emergency room or the ICU were analyzed (location: university hospital in Genoa, Italy).

### Animal study

After premedication with atropine, midazolam and ketamine, 12 pigs were anesthetized with continuous infusion of ketamine, atracurium and midazolam, intubated and mechanically ventilated in volume-controlled mode (VCV) in supine position with tidal volume set at 6 mL/kg (Evita Infinity, Dräger, Germany), under continuous hemodynamic and respiratory monitoring. Experimental ARDS was induced with a previously described double-hit model [17], consisting in warm saline solution lung lavages followed by injurious mechanical ventilation. Twenty-six hours after the induction of ARDS, three CT scans of the whole lung were performed in sequence during respiratory hold: (1) standard spiral scan with 180 mAs tube current-time product (Spiral, 180 mAs); (2) sequential scan with 1-mm slices interleaved by 20 mm feed and 180 mAs (Sequential, 180 mAs); (3) sequential scan with 1-mm slices, 20 mm feed and 50 mAs (Sequential, 50 mAs). The feed of 20 mm was chosen because we expected it to result in a number of about ten slices per scan, as in a previously described method [12, 13]. Respiratory hold was obtained setting the ventilator in continuous positive airway pressure (CPAP) mode, with the pressure level set equal to the mean airway pressure achieved during VCV; the three scans were interleaved by 1 min of VCV, to avoid desaturation. Images were acquired with a Biograph 16 PET/CT scanner (Siemens, Knoxville, US) with 120 kV tube voltage, collimation 16 × 0.75 mm (spiral scan) or 1 × 1 mm (sequential scans) and dose modulation (CARE Dose4D, Siemens, US); image reconstruction was performed with B70s convolution kernel, 1-mm slice thickness and 0.508 × 0.508 mm^2^ voxel size. The dose length product (DLP), scan acquisition time and CT dose index (CTDI) were extracted from the structured Digital Imaging and COmmunications in Medicine (DICOM) report, and dose exposure (E) was estimated using a previously published method [18]. At the end of the imaging acquisition, animals were killed with 2 g intravenous thiopental followed by 50 mEq KCl.

### Human study

To verify whether the findings of the animal study could be translated to humans, we retrospectively analyzed chest CT scans of critically ill patients admitted at the emergency department or at the ICU. The CT scanner was a LightSpeed 16 (GE Medical Systems, Milwaukee, US), tube current was 120 kVp, pitch factor 1.75 and collimation 16 × 0.625 mm. We included only scans of patients aged ≥18 performed with a standard protocol: no contrast medium, 1-mm slice interleave, 1.25 mm thickness and medium-soft convolution kernel. From spiral scans, subsets of images including the entire lung were extracted at increasing distance between slices, mimicking the feed in sequential scans, from 2 to 50 mm in 1-mm steps.

To assess translatability of the results obtained in the animal study, we observed the current-time product used in the clinical practice, the cranio-caudal scan length, and the reliability of the extrapolation from equally spaced 1-mm thin slices, as a proof of the geometric robustness of the extrapolation method to the anatomical differences in chest shape between pigs and humans.

### Image analysis

Lung segmentation was performed with ITK-SNAP (http://www.itksnap.org) with a semi-automated algorithm followed by manual refinement, image analysis with custom MATLAB scripts (MathWorks, MA, US). Lung aeration compartments were calculated as percent of the total lung weight using the following Hounsfield unit (HU) thresholds [19]: hyper-aerated (−1000 to −901 HU), normally aerated (−900 to −501 HU), poorly aerated (−500 to −101 HU), non-aerated (−100 to +100 HU). Total lung volume (TLV), weight (TLW) and aeration compartments were extrapolated from each sequential scan in pigs or subset of images in humans, readapting a previously reported formula [20]:

$$ Mlung={\displaystyle \sum_{i=1}^{N-1}}\left( f\cdot \frac{M_i+{M}_{i+1}}{2\cdot t}\right)+\frac{M_1+{M}_N}{2} $$

**Fig. 1:**
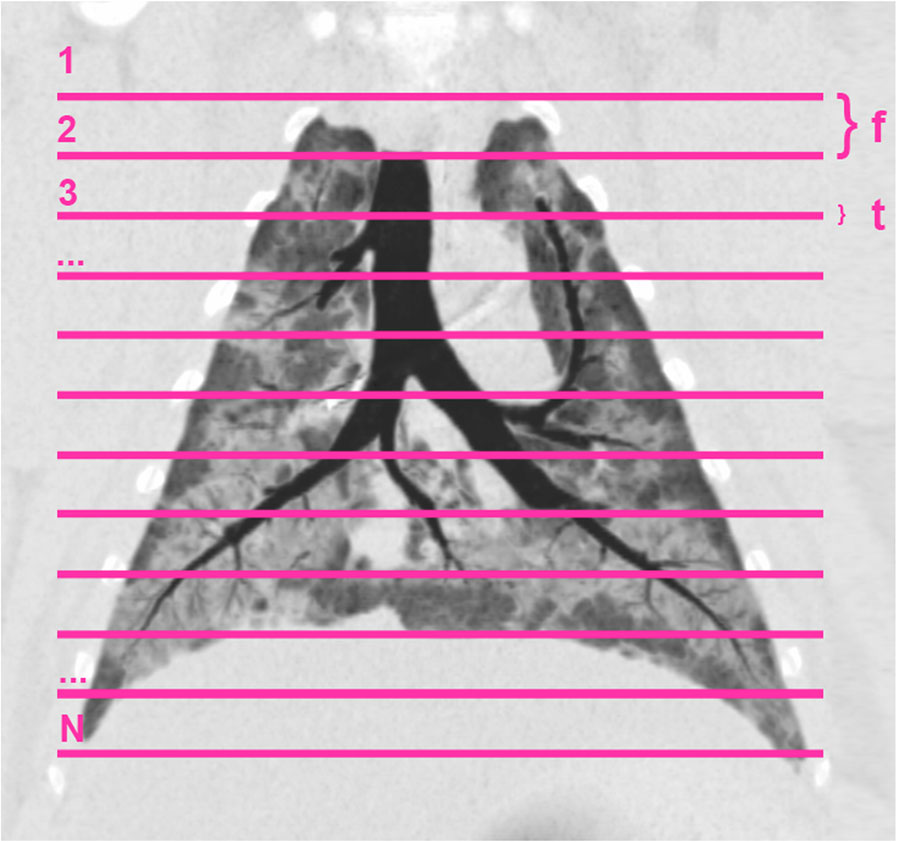
Coronal plane reconstruction of a representative animal. *Lines* represent 12 slices acquired with a sequential protocol using 1.0-mm slice thickness (*t*), interleaved by 20 mm feed (*f*)

Where *N* is the number of slices, *t* is the slice thickness, *f* the distance between slices (feed) and *M*_*i*_ the lung mass in the *i*th slice (see Fig. 1); the same formula was used for lung volume and aeration compartments.

### Outcome measures

The main endpoints were the effective dose (*E*) reduction and bias between sequential scans and the spiral reference scan. Post-processing workload was estimated as the number of slices that the operator must segment. Safety and feasibility were assessed measuring the scan acquisition time, ensuring that the duration of apnoea was below 40 s, namely the typical duration of a recruitment manoeuvre used in ARDS patients [21].

### Analysis plan

In the animal experiment, the Bland–Altman method was applied calculating bias and limits of agreement (LOA) for each variable to assess the agreement with the reference spiral scan. In the human study, data from each subset of images were compared with the entire spiral scan, using the same method. Comparisons of dose measures, number of slices and acquisition time were performed with a Wilcoxon or Friedman test with Dunn’s post hoc, as appropriate. Correlations were sought using Spearman’s *ρ*. Data are reported as median (interquartile range), where not specified otherwise. Sample size calculation was based on a previously published paper [10] and a pilot scan conducted on a CT dosimetry phantom: we needed to enrol at least four animals to achieve a 95% power to detect a dose reduction of at least 80%. Statistical analysis was performed with R version 3.2.3 (http://www.r-project.org) and significance assumed for *p* < 0.05.

## Results

### Animal experiment

Twelve pigs developed lung injury after eight saline lavages and 172 (115–218) min of injurious ventilation; at the time of the CT scan, they were ventilated in VCV mode, with 8.0 (5.0–8.0) cmH_2_O positive end-expiratory pressure and 0.4 (0.4–0.5) fraction of inspired oxygen, resulting in a PaO_2_/FiO_2_ ratio of 186.3 (179.5–253.5). The ventilation and gas exchange data refer to the time-point immediately preceding the transfer of the animal to the CT facility, namely 24 h after the induction of lung injury.

The results of the quantitative CT analysis and the Bland–Altman comparisons are reported in Table 1. The use of the dose modulation resulted in measured current-time product values slightly different from the pre-set ones: 169 (164–180), 132 (124–141) and 45 (43–49) mAs, respectively. The cranio-caudal length covered by sequential scans was 240 (220–260) mm.

**Table 1:** Prospective validation in pigs

CT Variable	Spiral, 180 mAs	Sequential, 20 mm feed, 180 mAs			Sequential, 20 mm feed, 50 mAs		
	Analysis results	Analysis results	*ρ*	Bias (LOA)	Analysis results	*ρ*	Bias (LOA)
Total lung volume (ml)	1184 (1143–1244)	1168 (1140–1270)	0.867	2 (−64–69)	1185 (1156–1286)	0.839	10 (−62 to 82)
Total lung weight (g)	665 (598–681)	650 (581–672)	0.902	−10 (−59–39)	658 (593–683)	0.727	−2 (−65 to 61)
Hyper-aerated tissue (%)	0.2 (0.1–0.4)	0.3 (0.1–0.5)	0.993	0.1 (−0.3–0.5)	0.3 (0.2–0.5)	0.993	0.2 (−0.4 to 0.7)
Normally aerated tissue (%)	26.8 (23.6–38.8)	27.4 (24.9–37.8)	0.986	0.4 (−2.7–3.5)	26.8 (23.3–36.9)	0.993	−0.1 (−3.0 to 2.7)
Poorly aerated tissue (%)	41.7 (32.9–45.6)	39.0 (30.2–45.1)	0.972	−1.9 (−4.9–1.1)	39.6 (30.5–45.3)	0.965	−1.8 (−5.6 to 2.1)
Non-aerated tissue (%)	29.3 (18.2–34.3)	30.0 (18.0–34.7)	0.972	1.4 (−3.3–6.1)	30.3 (18.6–35.8)	0.874	1.7 (−3.1 to 6.5)

Comparison between CT analysis performed on the reference spiral scan and extrapolated from the two sequential scan protocols. *ρ* is Spearman’s rank correlation between extrapolated variables and the reference ones. Data are medians (interquartile ranges). Bias (LOA) is the result of the Bland–Altman analysis, reported as bias (limits of agreement)

**Fig. 2:**
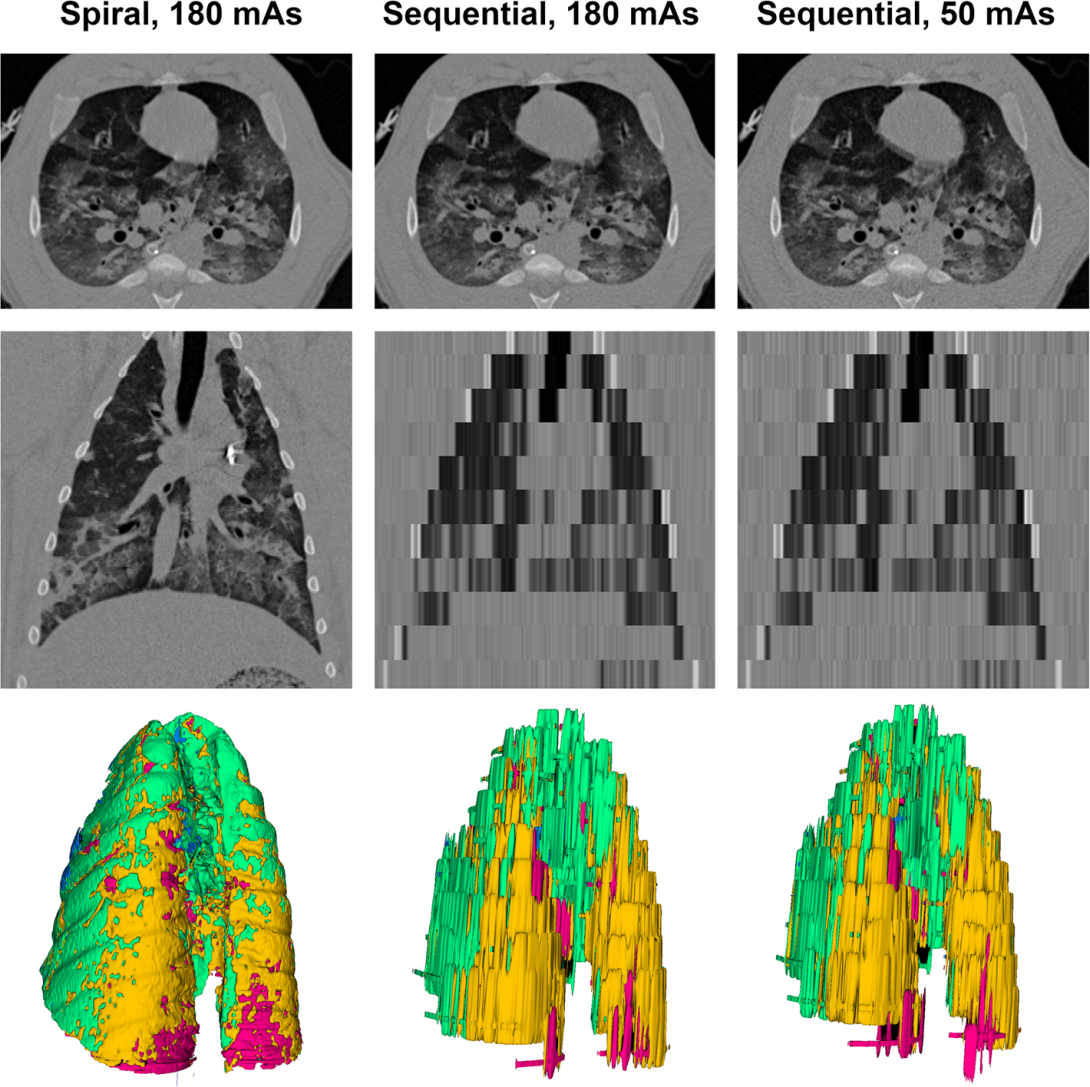
Scanning protocols. Axial (*upper panels*), coronal (*middle panels*) and 3D reconstructions (*lower panels*) of the three acquisition protocols in a representative animal. *Colors* in the 3D reconstruction represent clusters of lung tissue: hyper-aerated (*blue*), normally aerated (*green*), poorly aerated (*yellow*), non-aerated (*red*)

Both types of sequential scans resulted in a lower radiation dose, lower number of acquired slices and longer scan time, as shown in Table 2. Acquisition scan time was ≤33 s in all cases. The sequential scan at 50 mAs resulted in a median effective dose of 0.09, comparable to that of a digital chest X-ray [22]. Figure 2 illustrates axial, coronal and 3D reconstructions of the three acquisition protocols in a representative animal.

**Table 2:** Prospective validation in pigs

Parameter	Spiral, 180 mAs	Sequential, 20 mm feed, 180 mAs		Sequential LD, 20 mm feed, 50 mAs	
			*p*		*p*
CTDI (mGy)	4.4 (4.1–4.9)	0.46 (0.41–0.51)	0.029	0.18 (0.15–0.18)	<0.001
DLP (mGy·cm)	113.7 (103.2–138.6)	10.2 (9.5–12.6)	0.029	4.0 (3.4–4.7)	<0.001
E (mSv)	2.3 (2.1–2.8)	0.21 (0.19–0.26)	0.029	0.09 (0.07–0.10)	<0.001
Scan acquisition time (s)	6.8 (6.3–7.0)	22.7 (21.4–25.4)	0.004	24.1 (22.3–26.3)	<0.001
Number of slices	244 (227–252)	12 (11–13)	<0.001	12 (11–13)	0.001

Dosimetry, scan execution time and number of acquired slices in the three scan protocols in pigs. *P* values refer to the paired comparison with the reference spiral scan. *CTDI* computed tomography dose index, *DLP* dose length product, *E* effective dose. Data are median and (interquartile range)

### Human study

We collected CT scans from 32 critically ill patients, whose characteristics are reported in the Additional file 1: Supplemental digital content—eTable 1. Most patients (*N* = 30) were included in another unrelated study concerning the assessment of lung hyperinflation in critically ill patients [23], and the analyses included in this manuscript have not been published before. For each patient, 49 subsets of images at increasing distance between slices were extrapolated and compared with the whole spiral scan, for a total of 1568 comparisons. Bias and LOA worsened at increasing slice interleave (see Additional file 1: Supplemental digital content). The accuracy of extrapolation at 20 mm of distance between slices is shown in Table 3: the number of analyzed slices decreased from 250 (233–278) to 13 (12–14) (*p* < 0.001). The lung cranio-caudal diameter was 250 (234–276) mm, the number of slices analyzed in the subset with interleave of 20 mm was 12 (12–14), while the current-time product was 229 (201–303) mAs. These three findings were similar to those observed in animal scans, and to the values reported in a study describing the limits of normality of quantitative CT [8].

**Table 3:** Extrapolation method in humans

CT Variables	Spiral	Extrapolation 20 mm	ρ	Bias (LOA)
Total lung volume (ml)	3700 (2908–5327)	3736 (2896–5313)	0.997	6 (−60 to 72)
Total lung weight (g)	912 (754–1147)	903 (771–1155)	0.997	2 (−37 to 41)
Hyper-aerated tissue (%)	2.5 (0.1–9.1)	2.3 (0.1–9.1)	0.999	0.1 (−0.4 to 0.5)
Normally aerated tissue (%)	65.5 (50.1–77.1)	66.4 (49.5–77.2)	0.996	0.0 (−1.9 to 1.9)
Poorly aerated tissue (%)	15.0 (11.3–18.7)	15.4 (11.3–18.7)	0.982	0.1 (−1.1 to 1.4)
Non-aerated tissue (%)	8.0 (3.1–27.5)	7.1 (3.1–26.4)	0.986	−0.2 (−2.2 to 1.8)

Comparison between CT analysis performed on the reference spiral scan and extrapolated from a subset of slices interleaved by 20 mm (Extrapolation 20 mm). *ρ* is Spearman’s rank correlation between extrapolated variables and the reference ones. Data are medians (interquartile ranges). Bias (LOA) is the result of the Bland–Altman analysis, reported as bias (limits of agreement)

**Fig. 3:**
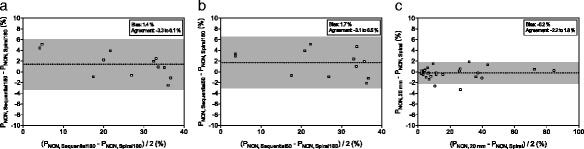
Bland–Altman plot for the non-aerated lung compartment. Comparisons are performed in pigs between the reference spiral scan and the sequential scan at 180 mAs (**a**) and 50 mAs (**b**), and in humans between the entire spiral scan and the extrapolation from slices interleaved by 20 mm in humans (**c**)

Figure 3 shows the Bland-Altman comparison for the non-aerated compartment between the experimental protocols and the reference scans in pigs and humans. The Additional file 1 contains extensive details on the analysis results of both human and animal scans.

## Discussion

The main findings of our study were that (1) the results of the quantitative aeration analysis performed on the sequential scans had a good agreement with that performed on the whole spiral scan, (2) an approach combining sequential scan and low current-time product results in an effective radiation dose comparable to that of a chest X-ray without affecting significantly the accuracy of the quantitative analysis and (3) this extrapolation method is applicable in CT scans in humans.

This is the first study, to our knowledge, in which lung CT quantitative analysis is extrapolated from a sequential scan with a low current-time product. This combines the advantages of low-dose CT, namely using a lower tube current, with the radiation exposure of only few equally spaced thin chest portions. We achieved a dose reduction around 97%, compared to the 70% described when low tube current alone is applied [9, 10]. The high radiation burden has limited the application of quantitative CT to critically ill patients: for instance, in recent studies in perioperative medicine, ethical concerns have forced researchers to use a single CT slice analysis [24] or magnetic resonance imaging [25]. Using a new approach combining low-dose CT and sequential scans importantly lowers the risk-to-benefit ratio of a quantitative lung aeration assessment to a probably acceptable level.

The method described in this paper is a prospective validation of a modified protocol derived from a previously published work [12–14], in which the authors used a fixed number of ten slices. Translating this in a prospective acquisition protocol would be difficult, as it would require the correction of the distance between slices according to the patient’s lungs’ sagittal diameter. The use of a fixed interleave between slices is easier to implement, as a standard sequential scan with a fixed feed can be used. While not often used in the clinical practice, sequential scan acquisition is available in most modern spiral CT scanners; therefore, this protocol can be easily used in other institutions using different scanners. Overall, there was good agreement between the quantitative analysis performed with the sequential protocols and the spiral scan, with all biases being below 2% for aeration compartments, 10 ml for total volume and 10 g for lung weight.

In the sequential scans, we used thin slices of 1 mm. Slice thickness is a known factor affecting the assessment of aeration compartments, in particular hyper-aerated tissue [23, 26, 27]. There is no unanimous consensus concerning which slice thickness should be preferred for the quantitative analysis of lung aeration [5]: several research groups used 5 mm [8] or 1 mm [17] slices. In spiral scans, thicker slices result in a lower number of images to be segmented to perform analysis, and this might explain why many research groups avoided using thinner slices. Incrementing the slice thickness in sequential scans would result in an increase of dose exposure, therefore jeopardizing the advantages of the sequential scan. Given the lack of a reference standard, we proposed the use of 1-mm slices to prioritize the patients’ dose exposure reduction. However, in specific research settings where other slice thicknesses are warranted, the bias in hyperaeration assessment introduced by the use of thinner slices could be corrected with a recently validated mathematical method [23, 28]. In the studies estimating CT aeration from ten equally spaced slices of 5 to 10 mm thickness, the extrapolation method showed comparable performances in pigs [13], sheep [13], ponies [14] and humans [12]. Based on these previous findings, we hypothesized that extrapolating quantitative analysis from thin 1-mm slices, interleaved by 20 mm, could be used regardless of the discrepancies in lung shape between pigs and humans. Therefore, no changes to the slice sampling protocol were made when switching from the animal study to the human translatability assessment. In this study, the bias and LOA of the extrapolated scans worsened proportionally to the distance between slices, both in pigs and humans: this suggests that the extrapolation method is robust to anatomy variations, as already suggested [12–14]. Dose exposure and number of acquired slices are proportional to the cranio-caudal lung diameter and to the current-time product, which in our study were comparable in pigs and humans

While prospective validation in humans is still warranted to definitively validate this method, the translatability of our findings to humans is supported by the following factors: (1) in humans, the accuracy of the extrapolation from 1-mm slices is excellent; (2) the tube-current product used in the clinical routine in chest spiral CT scans in our institution was comparable to that used in the reference scan of our animal study; (3) the cranio-caudal length covered by a lung CT in humans and pigs is comparable.

Other dose reduction protocols that have been proposed rely only on the reduction of the tube current [9, 10]. The dose reduction that can be achieved with this technique is about 70%, i.e. the patient receives approximately one third of the dose he would have received with a conventional CT. The ultra-low-dose protocol we describe reduces this value to less than one twentieth, approaching the typical effective dose of a digital chest X-ray [22]. Moreover, tube current reduction alone does not reduce the number of slices that the operator has to segment, or visually assess, to perform the image analysis, resulting in a post-processing workload equal to that of a conventional CT.

The main pitfall of using sequential scans is that the acquisition time increases significantly. In our animal study, the sequential CT scan took around 24 s, compared to the 7 s of the spiral scan. Since the best clinical and research practice is to acquire the images during a breath hold, this increase in scanning time could pose challenges in particularly unstable patients, where desaturation is likely to occur. However, all scans were faster than a typical sustained inflation recruitment manoeuvre performed in ARDS patients, which is often performed increasing the airway pressure for 40 s [21]. Nonetheless, newer CT scanners might acquire images even faster.

The present study has several limitations. First, the translatability of our results to human scans was inferred, but not systematically tested: a prospective validation in humans is warranted, as while the reduction of dose exposure should be that observed in pigs, its absolute value might slightly differ. However, the human cohort covered a wide range of lung loss of aeration extension; therefore, the validity of the extrapolation method should not have been affected. Second, the pig scans were performed in breath hold but interleaved by a short period of mechanical ventilation to avoid desaturation, without recruitment manoeuvres between scans: part of the bias between sequential and spiral scans might be explained by this; however, this would result in an over- rather than under-estimation of the bias. Third, human scans were collected retrospectively, with poor control on the acquisition parameters. In particular, intubated patients were scanned during uninterrupted mechanical ventilation and this could have increased motion artefacts. However, since comparisons were made within and not between patients, this should not affect the interpretation of the results of our study. Fourth, human CT scans were previously included in another research and were performed on clinical indication on subjects with either healthy or injured lungs; however, the amount of non-aerated tissue ranged between 1 and 80% therefore can be considered representative of a mixed population of critically ill patients with a broad spectrum of lung conditions. The generalizability of our findings should be tested on a prospective cohort of ARDS patients.

## Conclusions

In conclusion, the combination of low tube current and sequential acquisition of thin slices interleaved by 20 mm provides an acceptable estimate of lung CT quantitative analysis compared to the entire spiral scan, with a notable reduction in dose exposure in pigs. In human CT data, lung aeration analysis could be extrapolated from a subset of thin equally spaced slices, as in the animal study.

## Additional file

Additional file 1: Details on the analysis on pigs and human scans. (PDF 326 kb)

## Abbreviations

ARDS: Acute respiratory distress syndrome
CPAP: Continuous positive airway pressure
CT: Computed tomography
CTDI: Computed tomography dose index
DICOM: Digital Imaging and COmmunications in Medicine
DLP: Dose length product
HU: Hounsfield units
ICU: Intensive care unit
LOA: Limits of agreement
PET: Positron emission tomography
TLV: Total lung volume
TLW: Total lung weight
VCV: Volume-controlled ventilation
