# Supplementary material for: Ultra-low-dose sequential computed tomography for quantitative lung aeration assessment—a translational study
Source: Intensive Care Med Exp. 2017 Apr 4;5:19. doi: 10.1186/s40635-017-0133-6 (PMC5380570; doi:10.1186/s40635-017-0133-6)
Supplement: Additional file 1: — Details on the analysis on pigs and human scans. (PDF 326 kb) [file 40635_2017_133_MOESM1_ESM.pdf]

Ultra-low dose sequential computed tomography for quantitative lung aeration assessment: a translational study

Online Supplemental Materials

Lorenzo Ball, Anja Braune, Francesco Corradi, Claudia Brusasco, Alessandro Garlaschi, Thomas Kiss, Thomas Bluth, Francesca Simonassi, Alice Bergamaschi, Jörg Kotzerke, Marcus J. Schultz, Marcelo Gama de Abreu, Paolo Pelosi

TABLE OF CONTENTS

eTable 1: Patients' Characteristics 2

1. PROSPECTIVE ANIMAL STUDY 3

1.1 Results of the Bland Altman analysis on the CT variables as function of the distance between slices in pigs. 3

1.2 Bland Altman comparison between spiral scan (180 mAs) and sequential scan (20 mm feed, 180 mAs) in pigs 4

1.3 Bland Altman comparison between spiral scan (180 mAs) and sequential scan (20 mm feed, 50 mAs) in pigs 5

1.4 Number of acquired slices 6

1.5 Scan acquisition time 6

1.6 Dosimetry 7

1.6.1 Computed Tomography Dose Index 7

1.6.2 Dose Length Product 7

1.6.3 Effective Dose 8

2. HUMAN RETROSPECTIVE TRANSLATABILITY ASSESSMENT 9

2.1 Results of the Bland Altman analysis on the CT variables as function of the distance between slices in humans. 9

2.2 Bland Altman comparison between extrapolation of 20mm-spaced slices and the complete spiral scan in humans 10

eTable 1: Patients' Characteristics

Variable	Patient Data (N = 32)		
Age, y (IQR)	62 (40 – 71)		
Sex (%)	19 Male (59.4), 13 Female (40.6)		
Clinical indication for chest CT (%)	Acute respiratory failure<br>Trauma<br>Other	18<br>4<br>10	*(56.3)*<br>*(12.5)*<br>*(31.3)*
Mode of ventilation (%)	Mechanically ventilated (intubated)<br>Mechanically ventilated (tracheostomy)<br>Spontaneously breathing	9<br>4<br>19	*(28.1)*<br>*(12.5)*<br>*(59.4)*
Main lung CT finding (%)	Injury (ARDS, pneumonia, contusion)<br>With focal loss of aeration<br>With diffuse loss of aeration<br>COPD<br>Healthy	17<br>8<br>9<br>7<br>8	*(53.1)*<br>*(25.0)*<br>*(28.1)*<br>*(21.9)*<br>*(25.0)*

eTable 1. Patients' Characteristics. CT computed tomography, IQR interquartile range, COPD chronic obstructive pulmonary disease, ARDS acute respiratory distress syndrome.

1. PROSPECTIVE ANIMAL STUDY

1.1 Results of the Bland Altman analysis on the CT variables as function of the distance between slices in pigs.

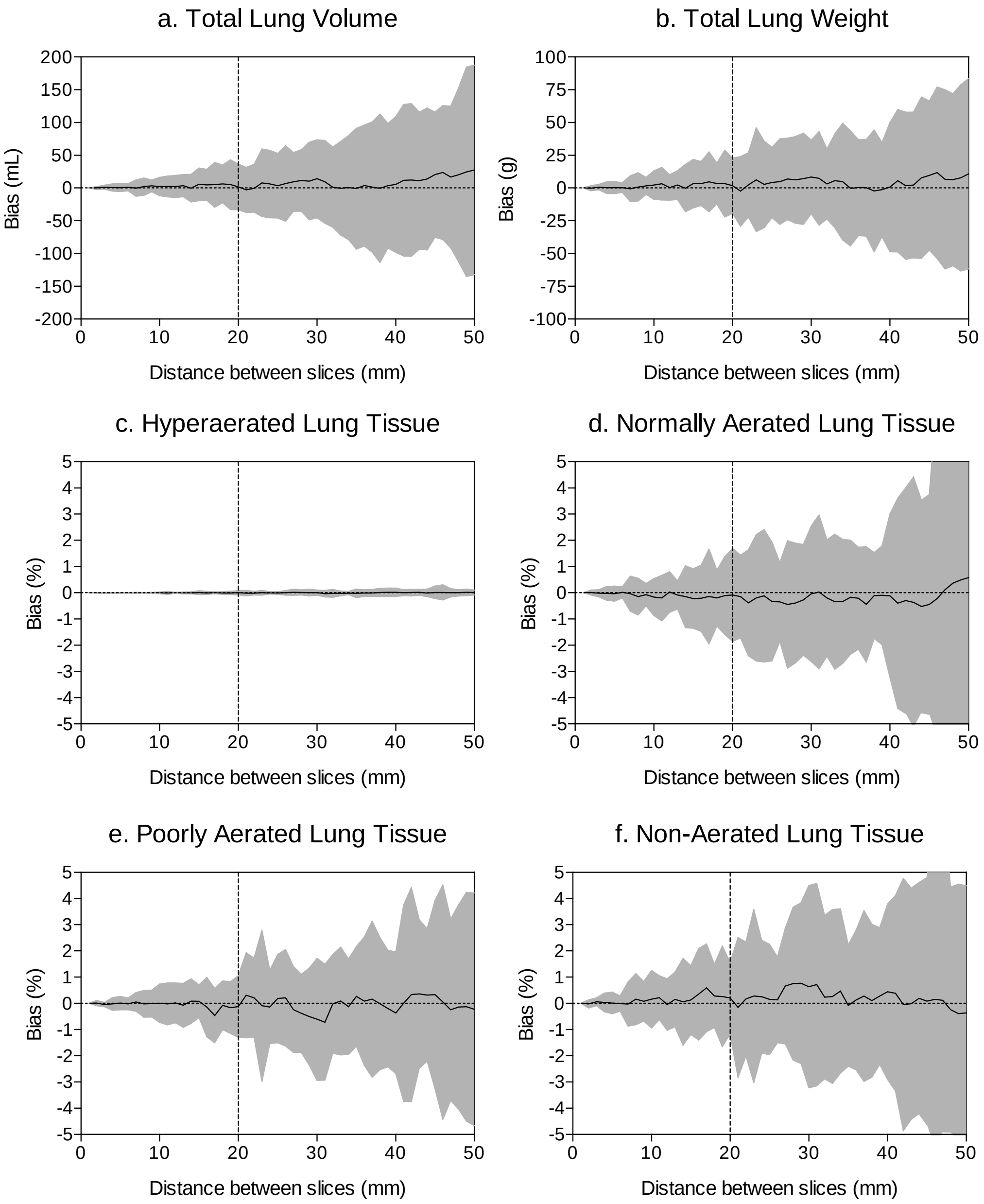

Black continuous line represents the bias between analysis extrapolated from image sub-sets and the reference spiral scan. Grey bands are limits of agreement (95% confidence interval of bias). The dotted vertical line represents 20 mm distance between slices, chosen for the sequential scans.

1.2 Bland Altman comparison between spiral scan (180 mAs) and sequential scan (20 mm feed, 180 mAs) in pigs

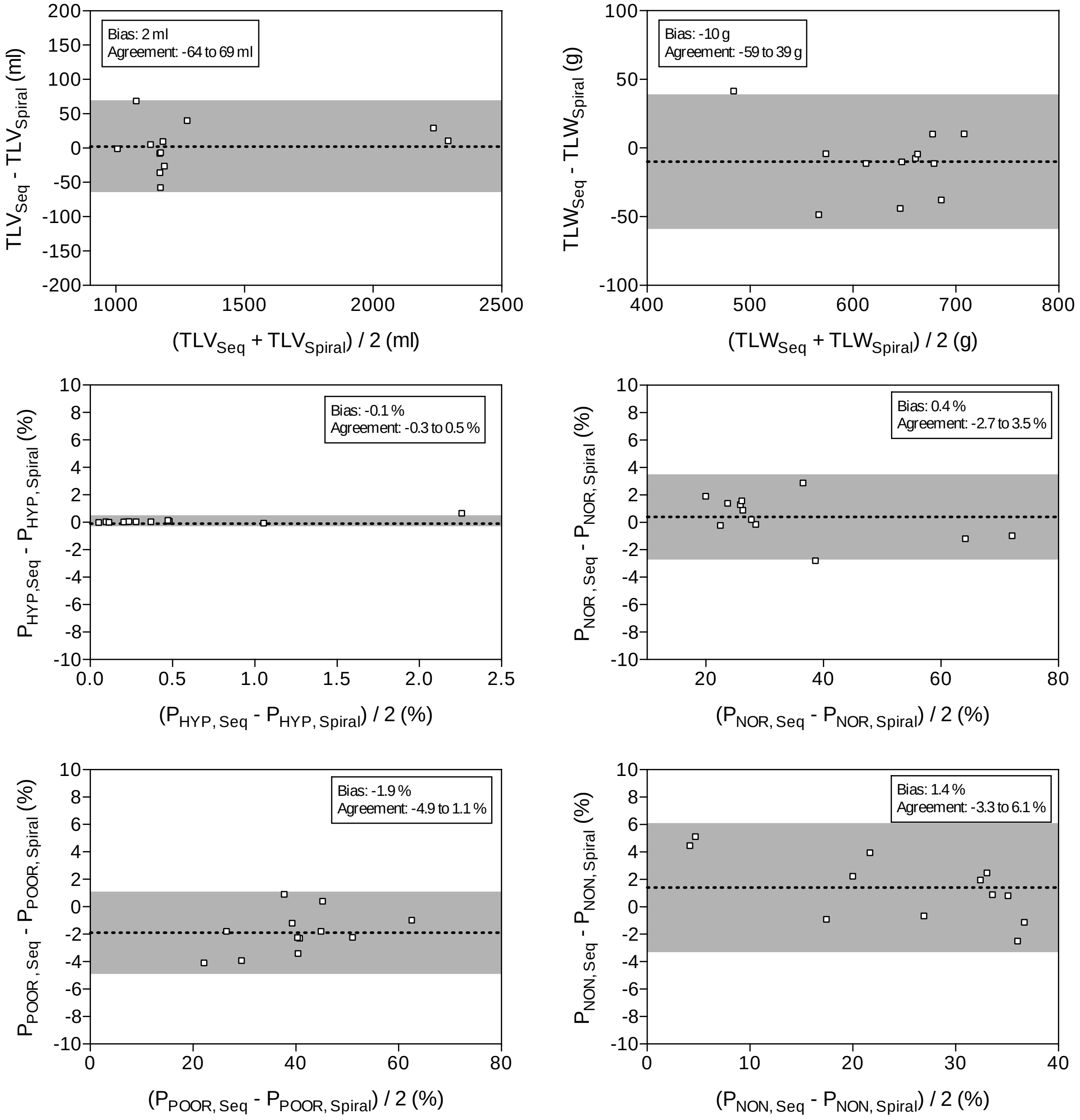

TLV: Total Lung Volume, TLW: Total Lung Weight, P_{HYP}: hyperaerated lung tissue %, P_{NOR}: normally aerated lung tissue %, P_{POOR} poorly aerated lung tissue %, P_{NON} non-aerated lung tissue %.

1.3 Bland Altman comparison between spiral scan (180 mAs) and sequential scan (20 mm feed, 50 mAs) in pigs

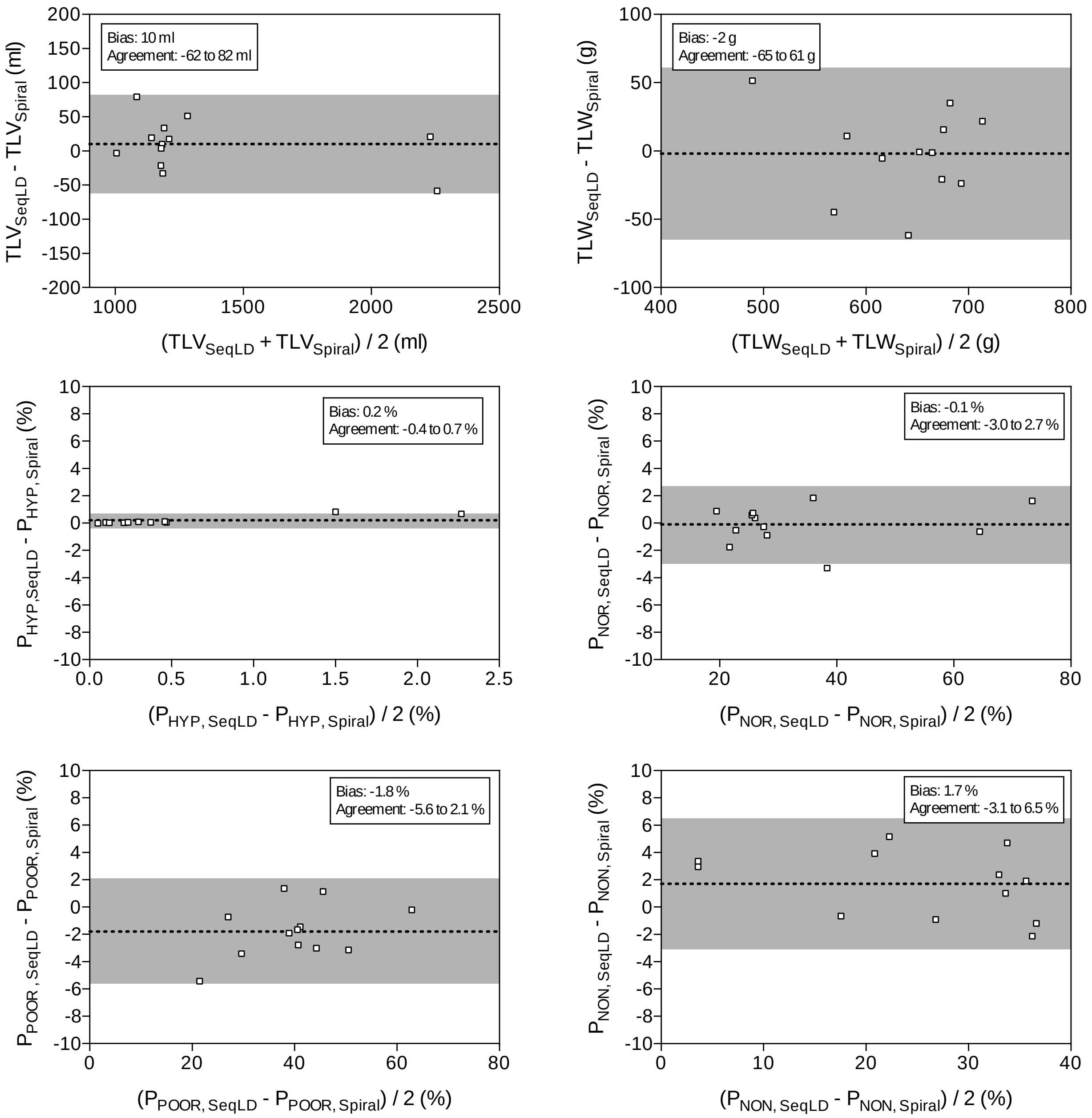

TLV: Total Lung Volume, TLW: Total Lung Weight, P_{HYP}: hyperaerated lung tissue %, P_{NOR}: normally aerated lung tissue %, P_{POOR} poorly aerated lung tissue %, P_{NON} non-aerated lung tissue %.

1.4 Number of acquired slices

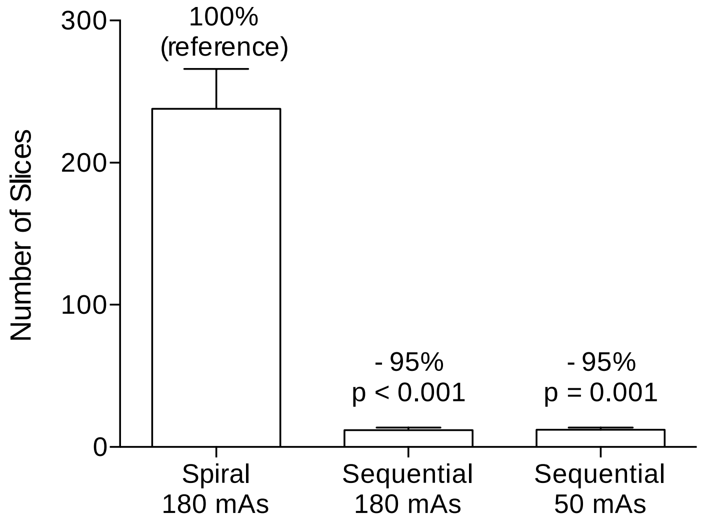

1.5 Scan acquisition time

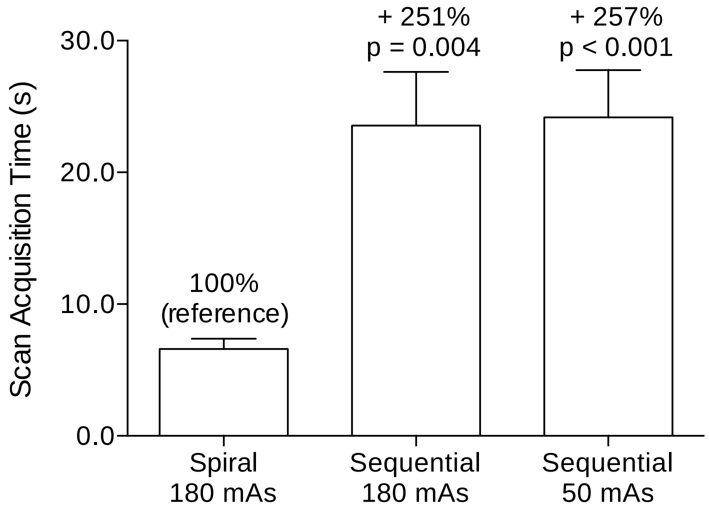

1.6 Dosimetry

1.6.1 Computed Tomography Dose Index

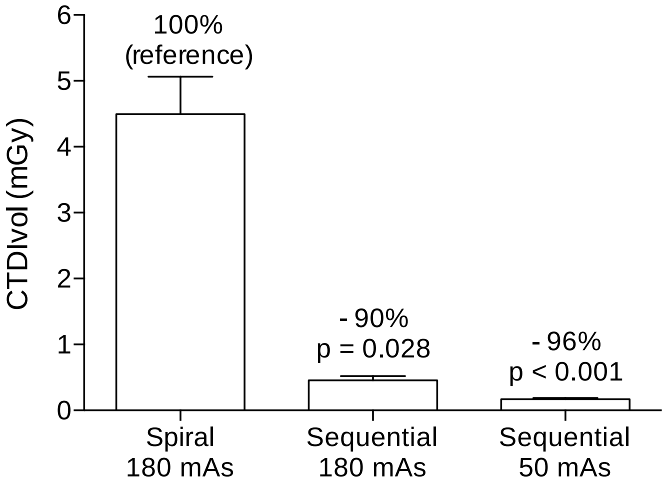

1.6.2 Dose Length Product

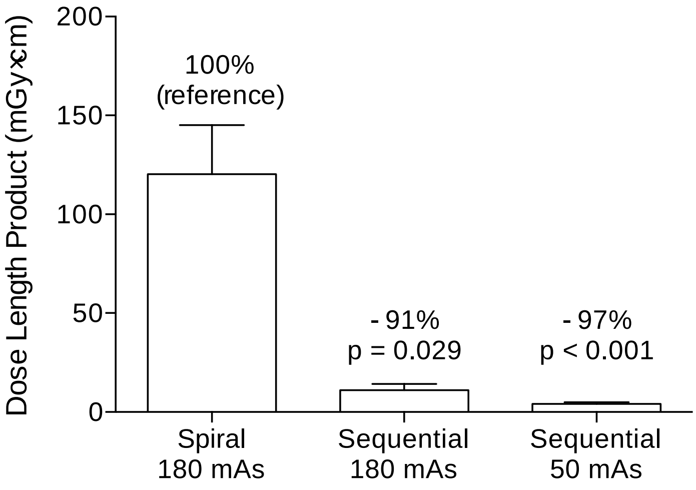

1.6.3 Effective Dose

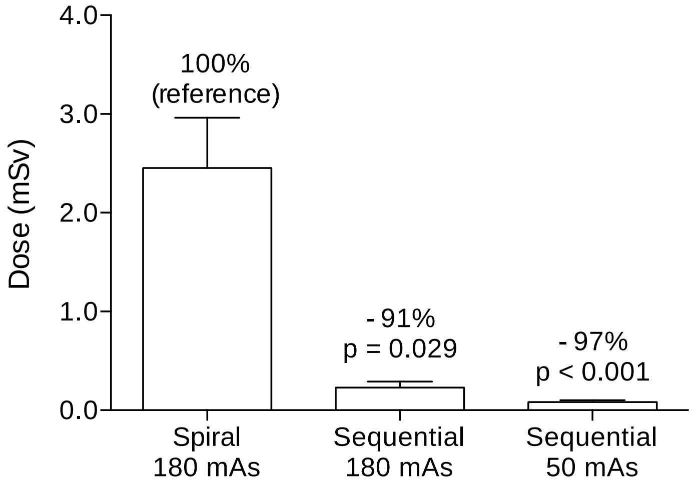

2. HUMAN RETROSPECTIVE TRANSLATABILITY ASSESSMENT

2.1 Results of the Bland Altman analysis on the CT variables as function of the distance between slices in humans.

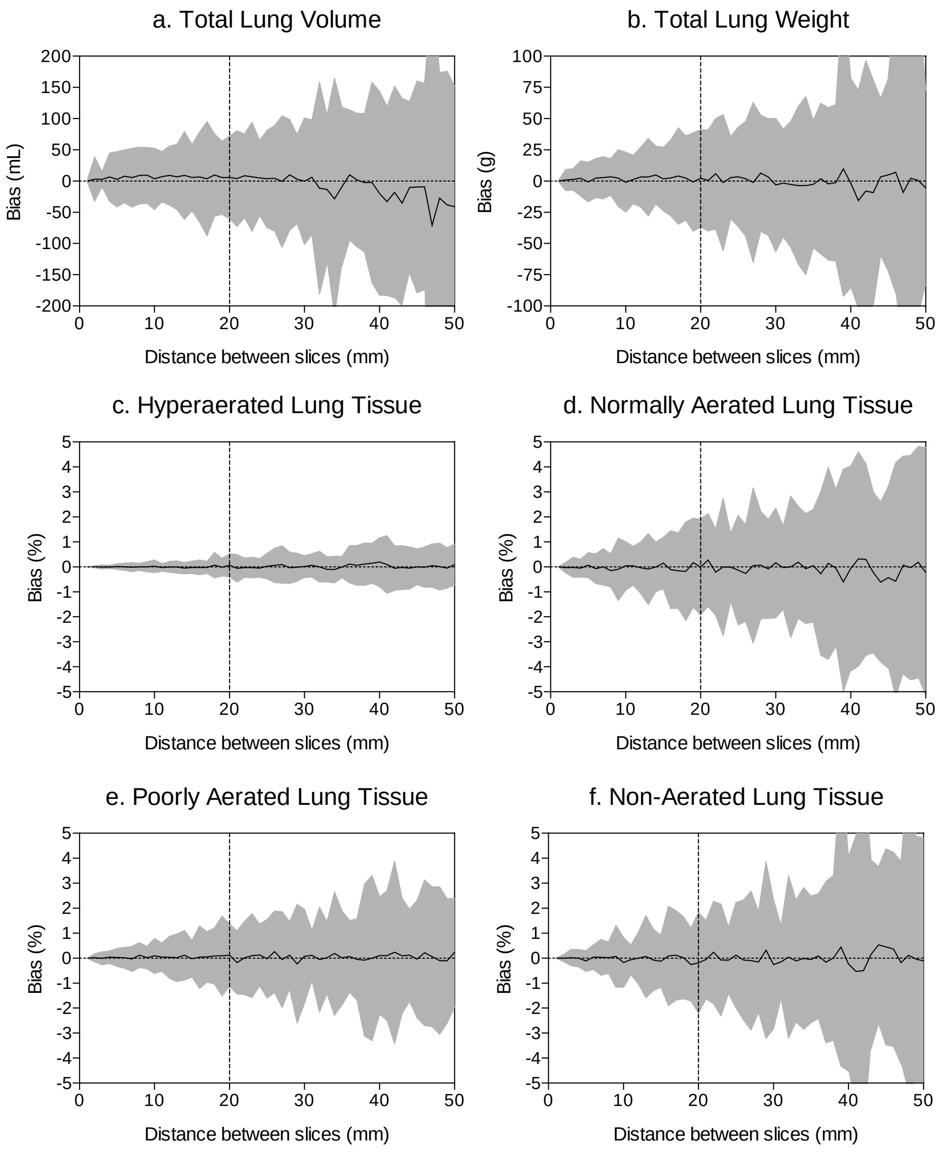

Black continuous line represents the bias between analysis extrapolated from image sub-sets and the reference spiral scan. Grey bands are limits of agreement (95% confidence interval of bias). The dotted vertical line represents 20 mm distance between slices, as used in the animal study.

2.2 Bland Altman comparison between extrapolation of 20mm-spaced slices and the complete spiral scan in humans

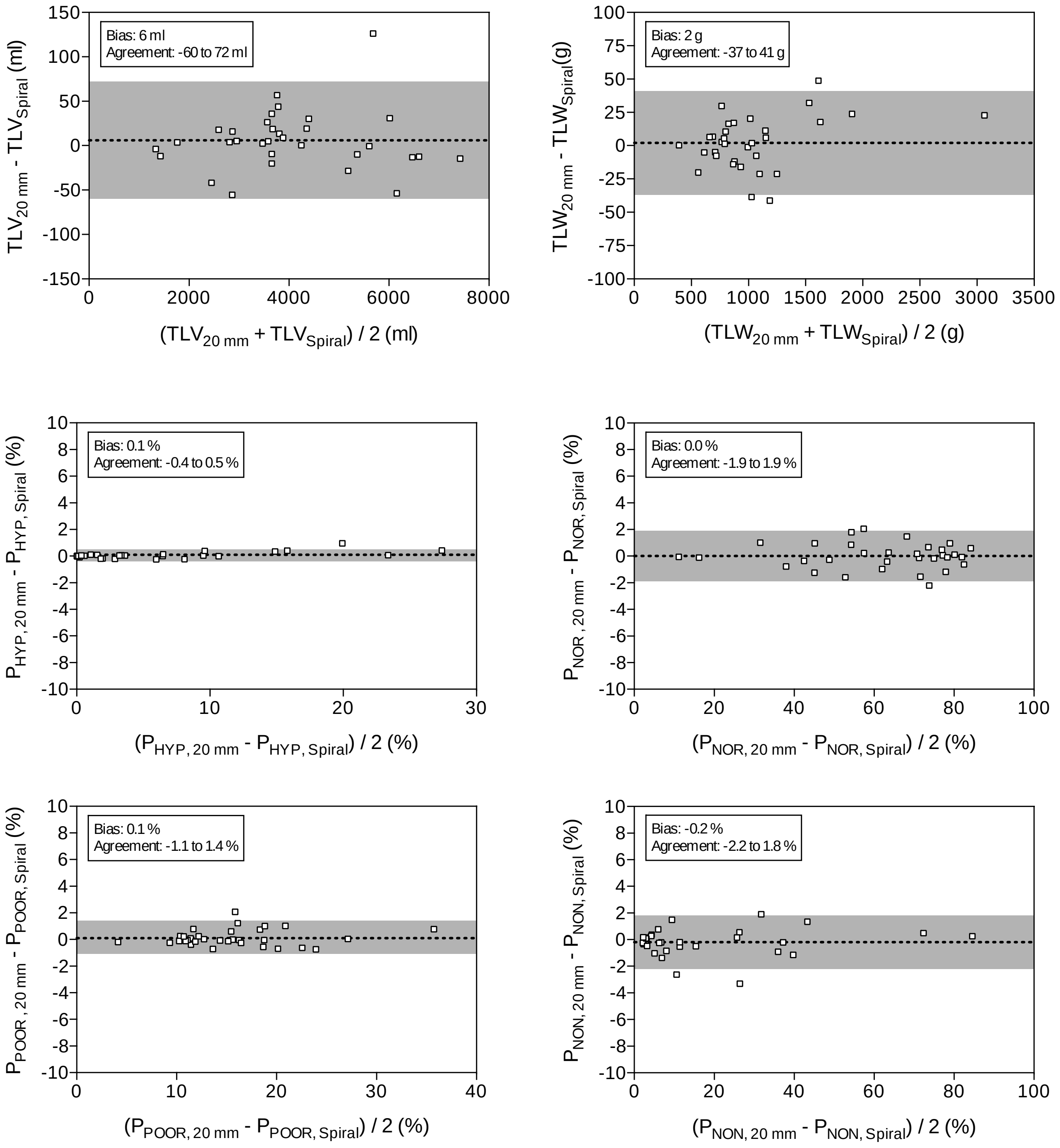

TLV: Total Lung Volume, TLW: Total Lung Weight, P_{HYP}: hyperaerated lung tissue %, P_{NOR}: normally aerated lung tissue %, P_{POOR} poorly aerated lung tissue %, P_{NON} non-aerated lung tissue %.
